# 878. Telemedicine Implementation in a Midwestern HIV Clinic: One Year Outcomes

**DOI:** 10.1093/ofid/ofab466.1073

**Published:** 2021-12-04

**Authors:** Nichole N Regan, Laura Krajewski, Nada Fadul

**Affiliations:** 1 Nebraska Medicine, Omaha, Nebraska; 2 University of Nebraska Medical Center, Omaha, Nebraska

## Abstract

**Background:**

During the COVID-19 pandemic, we realized the importance of limiting in-clinic interactions with patients who were stable on antiretroviral therapy to promote social distancing. Our HIV clinic adopted telemedicine practices, in line with the HHS Interim Guidance for COVID-19 and Persons With HIV. Several HIV clinics reported lower viral suppression rates during the pandemic. We aim to describe the implementation process as well as year one outcomes of telemedicine at our clinic.

**Methods:**

In March 2020, we created telemedicine protocols; we also designed and continuously updated algorithms for determining patient eligibility for telemedicine based on recent viral loads and last clinic visit. We monitored outcomes through electronic medical record chart reviews between May 1, 2020, and April 30, 2021. We collected patient demographics, and federal poverty level (FPL) information. We collected baseline and post-intervention rates of viral load suppression (VLS, defined as HIV RNA < 200 copies per mL), medical visit frequency (MVF, defined as percentage of patients who had one visit in each 6 months of the preceding 24 months with at least 60 days between visits) and lost to care (LOC, no follow up within 12 months period).

**Results:**

We conducted a total of 2298 ambulatory medical visits; 1642 were in person and 656 (29%) were telemedicine visits. Out of those, 2177 were follow up visits (649, 30% telemedicine). There was no difference of telemedicine utilization based on race (28% in African Americans vs. 32% in Whites); ethnicity (30% in Hispanic vs. 30% in Hon-Hispanic); gender (24% in females vs. 30% in males); or FPL (28% in FPL < 200% vs. 31% in FPL >200%). By the end of April 2021, overall clinic VLS rate was 94%, MVF was 48%, and there were 40 patients LOC compared to 92%, 49%, and 43 patients in April 2020, respectively.

**Conclusion:**

Telemedicine was a safe alternative to routine in-person HIV care during the COVID-19 pandemic. We observed similar rates of utilization across demographic and FPL status. Applying selection criteria, viral suppression and retention in care rates were not adversely impacted by shift to telemedicine modality.

**Disclosures:**

**All Authors**: No reported disclosures

